# Development of custom lead shield and strainer for targeted irradiation for mice in the gamma cell chamber

**DOI:** 10.1038/s41598-021-93964-5

**Published:** 2021-07-15

**Authors:** Nurhaslina Hasan, Nur Fatihah Ronny Sham, Muhammad Khalis Abdul Karim, Syed Baharom Syed Ahmad Fuad, Narimah Abdul Hamid Hasani, Effat Omar, Mohammad Johari Ibahim

**Affiliations:** 1 Faculty of Medicine, Universiti Teknologi MARA, 47200 Sungai Buloh, Selangor Malaysia; 2 Faculty of Dentistry, Universiti Teknologi MARA, 47200 Sungai Buloh, Selangor Malaysia; 3 Faculty of Science, Universiti Putra Malaysia, 43400 Serdang, Selangor Malaysia

**Keywords:** Radiotherapy, Targeted therapies, Biological physics

## Abstract

We presented a development of a custom lead shield and mouse strainer for targeted irradiation from the gamma-cell chamber. This study was divided into two parts i.e., to (i) fabricate the shield and strainer from a lead (Pb) and (ii) optimize the irradiation to the mice-bearing tumour model with 2 and 8 Gy absorbed doses. The lead shielding was fabricated into a cuboid shape with a canal on the top and a hole on the vertical side for the beam path. Respective deliveries doses of 28 and 75 Gy from gamma-cell were used to achieve 2 and 8 Gy absorbed doses at the tumour sites.

## Introduction

More than half of the patients with cancer have been treated with radiotherapy during their treatments either as adjuvant or neo-adjuvant^1^. Various studies radiotherapy can alter immune parameters in the tumour microenvironment^2–4^, that speculated has an impact on the cancer progression such as the abscopal effect. Therefore, the radiobiological study using an animal model is unavoidable to provide a better understanding and open for advancement in radiotherapy as one of the basic cancer therapies. This in vivo model has been extensively used in normal and tumour tissue radiobiology study which generates significant data for radiation dosimetry which requires less cost and most importantly the safety issue^5^.

We are currently investigating the effect of radiotherapy on mouse bearing tumour model as we are aware that only the tumour area needs to be exposed to the radiation. We found that it is challenging to irradiate the mice since hospital radiotherapy are unsuitable, whereas the commercially available for small animal irradiators such as SARRP (Xstrahl Inc., Swanee, GA, USA) and XRAD225Cx (PXI North Branford, CT, USA)^6^ are costly and unavailable in this region. Thus, the available solution for mice irradiation is to use a gamma cell irradiator (gamma-cell chamber) that employs radioactive isotope Cobalt-60 or Cesium-137.

There are numbers of application using gamma irradiation such as food sterilization, medical devices sterilization, irradiation of tissue culture plants and genetic study. In addition, the gamma-cell is purposely for laboratory study that embodied in different field including in medical. The nature of the gamma-cell cylindrical chamber is a geometric shape, which allows gamma-ray to be emitted into the entire chamber’s space from the wall. This emission will expose the samples with the gamma-ray. Since the intensity of gamma-ray from the gamma-cell is homogeneous in the chamber, there would be a disadvantage for radiotherapy study as the animal samples such as mice might be irradiated to a whole organ. The main purpose of radiotherapy is to irradiate the target volume which is on the selected region only. Hence, the development of an animal strainer with the lead shielding will help in irradiation on the selected part of the body and protect the healthy tissue at risk.

Lead is well-known for its capacity to provide a great photon shielding because of it high atomic number materials and has a good shielding properties. There is no comprehensive study on the custom-made radiation shield for gamma-cell irradiation that could specifically irradiate certain region. Generally, radiation shielding is used to protect non-interest region while allowing main exposure to the the targeted area. The magnitude of exposure then is monitored using Gafchromic EBT film as it has a high sensitivity and good linearity*.* Therefore, the objective of this study was to design and develop a custom-made shield made up from the lead to allow radiation dose delivery targeting the absorbed dose at 2 and 8 Gy to a particular area while protecting the surrounding healthy tissue of mouse bearing tumour model.

## Results

### Fabrication of lead shield

**Figure 1:**
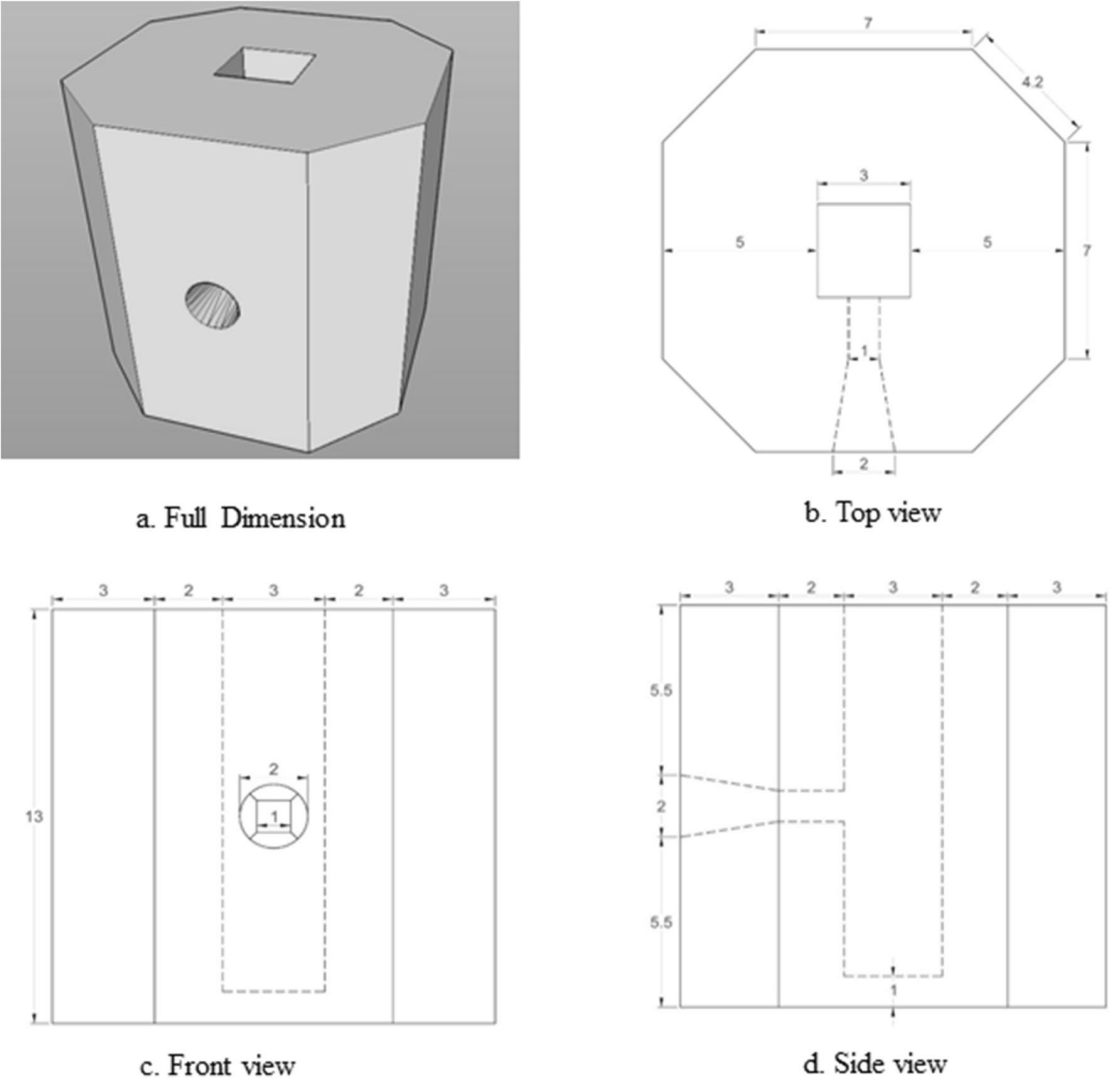
The shape of the lead shield in different views angle (**a**) Full dimension (**b**) Top view (**c**) Front view (**d**) Side view (Scale in cm).

Based on gamma shielding calculation, for 1.3 MeV energy of gamma-ray from Co^60^ source with 1.133 kGy/ hour, the lead shielding requires a thickness of 5 cm for reducing one-tenth of the dose rate. This shielding was molded in cuboid shape with a canal on the superior to load the mice in a strainer. At the lead shield lateral side, a hole was made for a gamma-ray beam path to radiate the tumour (Fig. 1a). The overall dimension of this shield is (1) The thickness of the cuboid is 5 cm from the canal and the height is 12 cm (2) The dimension of the canal for the mouse strainer is 3 cm × 3 cm with 12 mm depths from the top (Fig. 1b) and (3) The opening diameter of the beam path is 2 cm that shallow to 1 cm × 1 cm when reaching the canal (Fig. 1c,d).

### Mouse strainer

**Figure 2:**
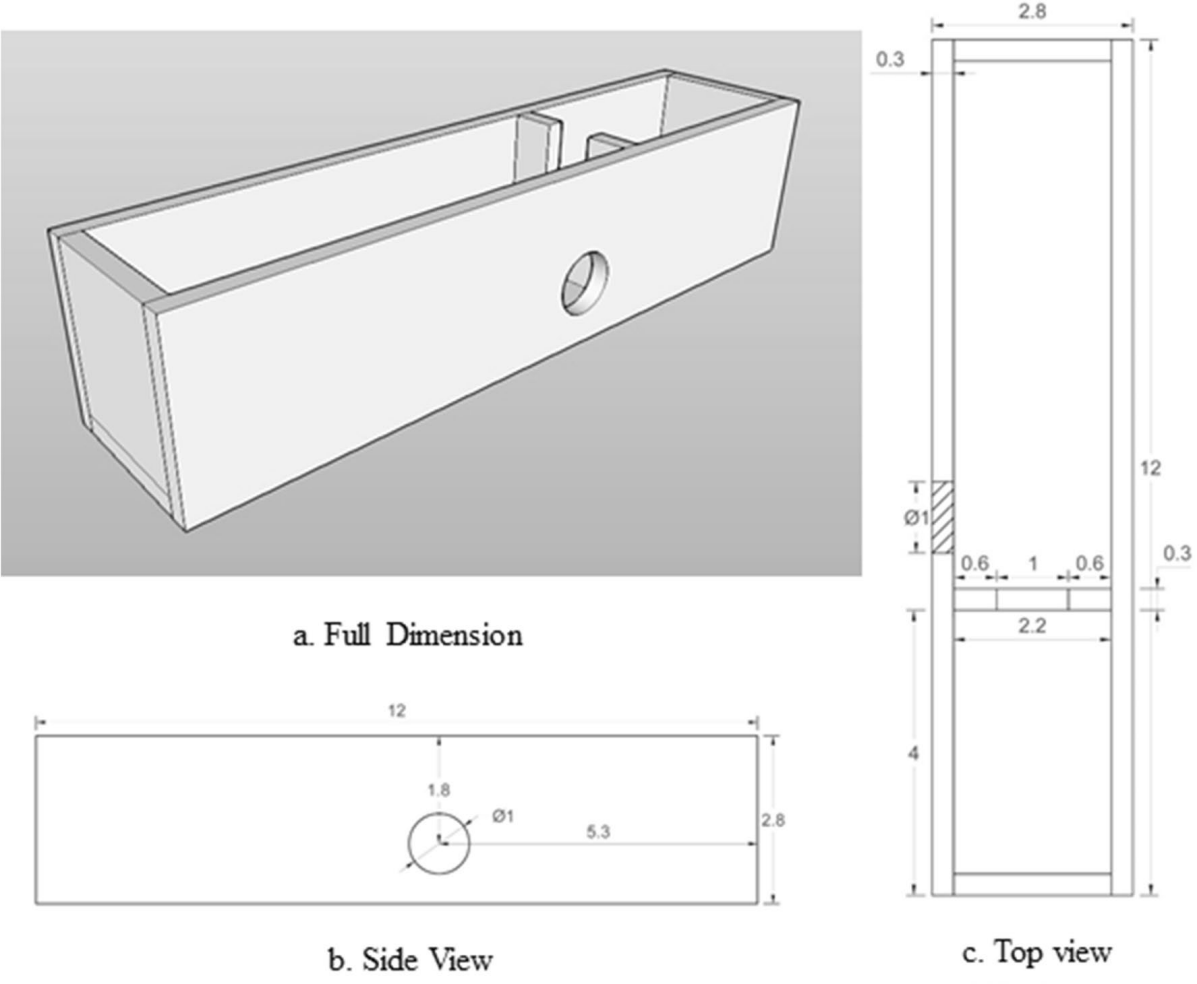
The shape of the mouse strainer in different views angle. There is a hole (1 × 1 cm) for the beam path. (**a**) Full dimension (**b**) Side view (**c**) Top view. (Scale in cm).

While loading the mouse into the lead’s canal, a customize strainer was used with the dimension is 2.8 cm × 2.8 cm with a height of the strainer of 12 cm (Fig. 2a–c). This strainer is made up of Perspex glass with a thickness of 3 mm and equipped with a 1 cm (diameter) hole at the side of the strainer’s wall. The dimension of this strainer was designed to hold the mouse in an upright position and ensure the tumour’s position was parallel to the lateral hole of the shield so that the beam path is precisely irradiated with additional usage as ventilation canal.

### Mice irradiation

**Figure 3:**
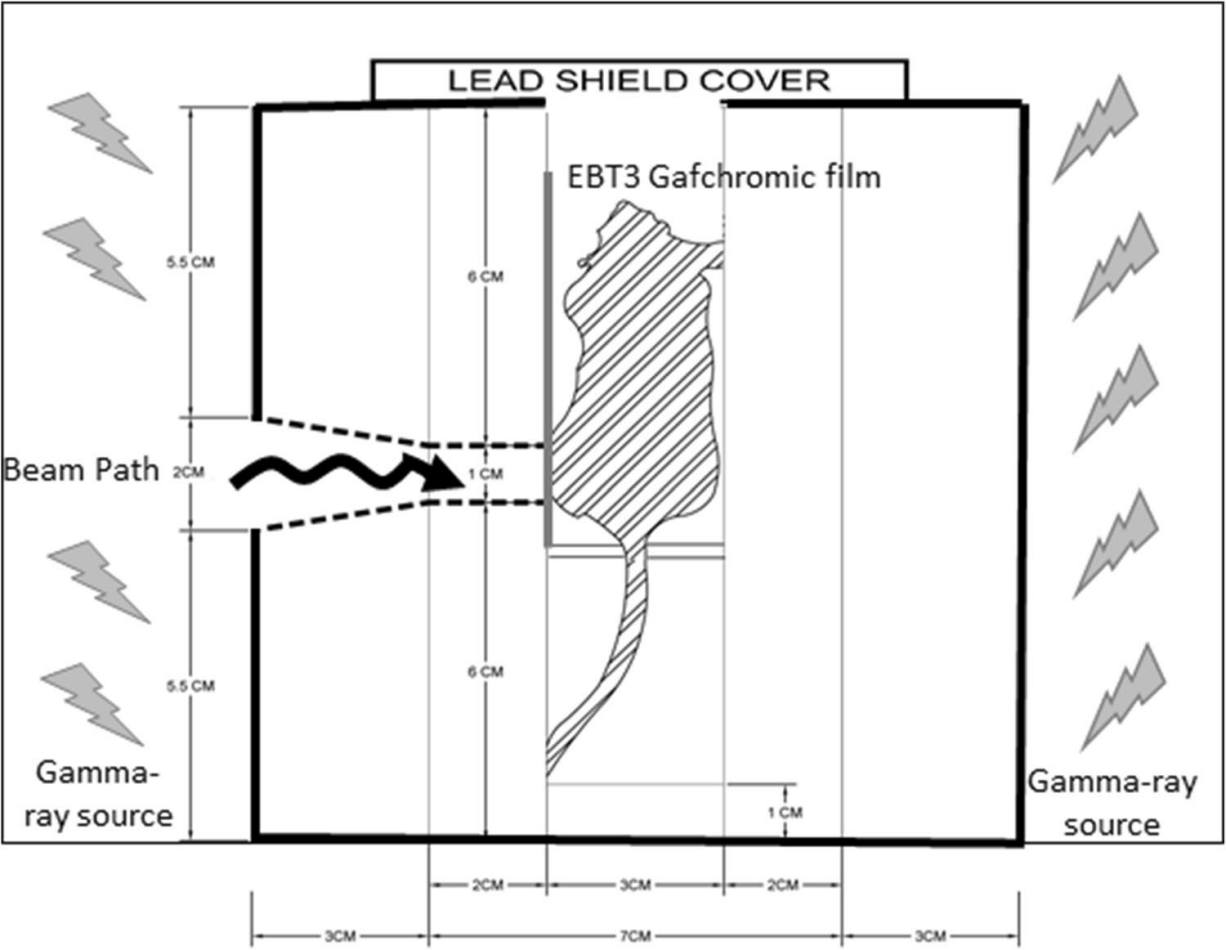
Illustration of cross-sectional from the lateral side of the lead shield in the gamma cell chamber from Co^60^ source (Scale in cm).

The irradiation process begins with the mouse bearing tumour inoculated at the left hind leg (Supp. Fig. S1a) was anesthetized with a mixture of ketamine and xylazine and placed in the strainer (Supp. Fig. S1b.). Mice were loaded in the mice strainer where the tumour position was adjusted directly to the beam path and EBT Gafchromic film was attached (Supp. Fig. S1c,d). The mouse is placed in mouse strainer and loaded in an upright position into the lead’s canal using the customize strainer (Supp. Fig. S2a). Then, the lead shield consisting of a mouse in the strainer was transferred into the gamma cell chamber for irradiation (Supp. Fig. S2b). The irradiation duration ranged from six seconds to three minutes, depending on the dose chosen (8 Gy to 105 Gy). We monitored the absorbed dose of gamma-ray using EBT3 Gafchromic film. Figure 3 showed the schematic diagram of the irradiation of mice in the gamma-cell chamber where the targeted area for irradiation is exposed through-beam path. The remaining body structure of the mice is protected from the radiation dose or received less irradiation dose. Therefore, the normal cells were protected from injury during the irradiation.

### Gamma cell dose calibration using EBT3 Gafchromic film

**Figure 4:**
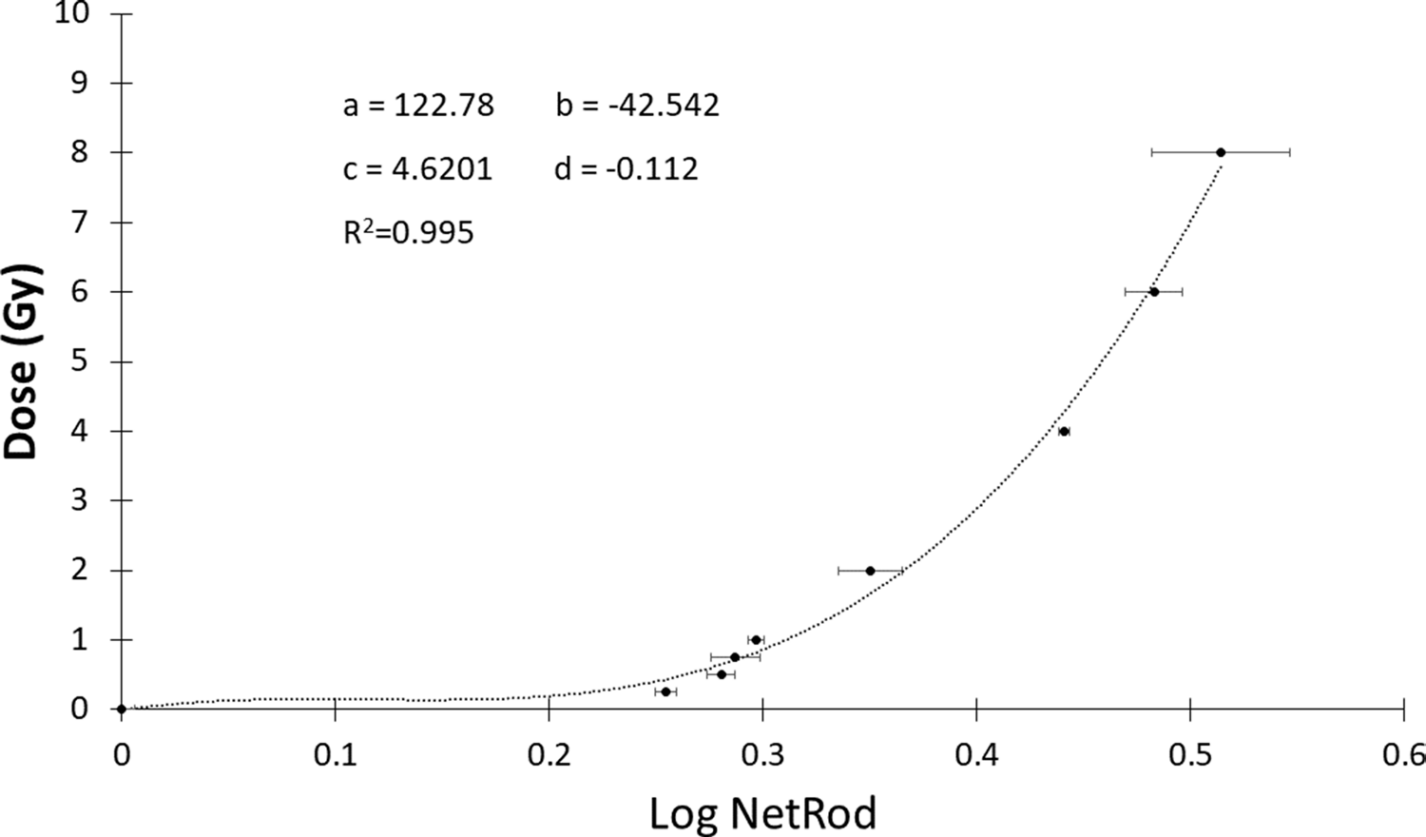
The calibration curve of gamma cell dose using polynomial power of 3. (Data n = 2, mean ± standard deviation). The calibration curve equation obtained is y = 122.78x^3^ + (− 42.542) x^2^ + 4.6201 + (− 0.0112).

Gamma-cell dose calibration was done using EBT3 Gafchromic film and NetRod formula in field size of 2 × 2 cm^2^ within the dose range of 0 to 16 Gy in triplicate for each energy. The NetRod value was analysed according to Equation 1 (in the methodology under section Film Digitation and Analysis). The RGB (red, green, and blue) channels were analysed and a graph of dose versus log NetRod value was constructed. The calibration curve was fit in RGB channel using polynomial power of 3 since it provided the best fitting curve (R^2^ = 0.995) (Fig. 4) compared to the other channels (graph not shown for other channels).

### Absorbed dose at the targeted tumour area

The absorbed dose at the tumour area and its surrounding were measured using Gafchromic film (Supp. Fig. S3). Based on the equation obtained from the polynomial power of 3, the absorbed dose was determined using different dose deliveries ranging from 30 to 90 Gy, as shown in Table 1. The dose deliveries were chosen based on our preliminary data. Here, we concluded that to achieve a 2 Gy absorbed dose, 28 Gy dose delivery is required, while for 8 Gy absorbed dose, 75 Gy dose delivery is required. We proceed to investigate the absorbed dose of 28 Gy and 75 Gy dose delivery using the mouse bearing tumour model. The results were presented in Table 2.

**Table 1:** The absorbed dose in the beam path from different delivery dose.

Dose delivery from Gamma-cell (Gy)	Dose absorb (Measure by EBT Gafchromic Film) (Gy)	
	Mean	Standard deviation
30	2.13	0.12
32	2.56	0.05
34	3.06	0.24
70	6.84	0.94
80	11.45	1.45
90	13.26	0.87

n = 2.

**Table 2:** The absorbed dose from 28 and 75 Gy delivery dose.

Mice No	Absorbed dose (Gy) from 28 Gy dose delivery	Absorbed dose (Gy) from 75 Gy dose delivery
1	2.03	8.24
2	2.18	8.77
3	1.84	8.84
4	1.94	9.28
5	2.12	8.82
6	2.23	8.97
Mean	2.06	8.82
Standard deviation	0.15	0.34

## Discussion

In the preclinical research, mouse bearing tumour model was frequently used to study the effect of radiation^7^. However, due to several limitations, including the high-cost to purchase a commercial irradiator specific for small animals such as mice, other sources of irradiator such as gamma-ray sources from the gamma cell chamber were utilized. The weighting of gamma rays is similar to X-ray and suitable for cancer treatment. Compared to a charged particles such as alpha and beta radiation, the gamma-ray can travel the farthest and has more penetrating power. There are various side-effects of gamma-ray irradiation has been reported on the small animal which includes liver toxicity^8^, lung damage^9^ and the worst is gene instability that generally contributes to pro-tumourigenic activity. Therefore, to minimize this pathological effect on the non-cancerous tissues, based on gamma shielding calculation, for 1.3 MeV energy of gamma-ray from Co^60^ source with 1.133 kGy/ hour, the lead shielding requires a thickness of 5 cm for reducing one-tenth of the dose rate. Up to date, there are several different custom-made lead shields have been developed to suit different xenograft model such as orthotopic and flank^7^; glioblastoma^10^, mouse flank^11^, and breast cancer shielding device^12^. All these shields are not only developed based on different models but also based on types of irradiator, either X-ray^7,11^ or gamma-ray^10^.

One of the main factors in designing this customize shield is the shape or the dimension of the radiation source. As in this present study, this dimension was designed to fit into the gamma-cell cylindrical chamber that enables only one mouse to be loaded into the shield, unlike the customized shield designed by Selvaraj et al.^13^ that will allow up to 5 mice to receive the treatment at once. Furthermore, we developed and used our custom-made lead shielding model in the gamma cell chamber to target the irradiation on the tumour site and protect the non-cancerous tissue*.* Our lead shielding model was designed to enable the gamma-rays emitted to pass through the beam window to the target area. The dose rate was chosen to acquire the desired absorbed dose. So higher delivery dose is required to achieve the absorbed dose. One of the factors that need to be considered while designing this custom-made strainer was the duration of the radiation treatment as this may give an impact on the studied animal such as hypoxia. In the present study, as the lateral hole of the strainer is designed to be perpendicular with the shield’s hole to ensure the precise irradiation towards the tumour area, it has also served as ventilation canal for the mice that suit to the treatment duration which takes less than 5 min.

The absorbed dose in the beam path is measured using the EBT3 Gafchromic film. There are several methods to measure the absorbed dose such as thermoluminescence dosimeter (TLD), Gafchromic film, and optically stimulated luminesce (OSL)^7^. EBT3 Gafchromic is the new generation of Gafchromic film. It measured the unknown dose based on the calibration of the standard curve. Soliman et al.^14^, suggested that EBT3 provided more accurate results compared to TLD while Howard et al.^15^ indicated that EBT Gafchromic film provided more advantages to measure radiation dose. However, according to Najafi et al.^16^, different findings were obtained on the dose measurement using Gafchromic film were due to the different factors such as the method for film digitation, the type of calibration curve used, or the uncertainty factor of gamma cell dose might cause deviation of the absorbed dose measurement. It can be seen in our data for delivery dose 28 Gy and 75 Gy that produce different readings from 6 replicates. Gan et al.^7^ recommended that the protocol for dose measurements should be standardized for each time of measurement by appropriate optimization established on the specific animal model and the source of radiotherapy.

Based on the Gafchromic film, we found that the exposure of gamma-ray at the abdomen and head of the mice in the lesser amount. A similar finding was obtained by Schleich and Herst^10^ that used a custom-made lead shield to irradiate glioblastoma cancer cells in the brain that found the radiation dose also spread to the abdomen and the leg of the rat with a lesser amount. It proves that our lead shield can reduce radiation dose to healthy tissue. However, the significant limitation of our model is the weight of the lead shield (approximately 25 kg) and required careful handling to transfer it into the gamma cell chamber during irradiation. Lead is also considered hazardous and high in toxicity which requires a safety measure to reduce the potential risk during the fabrication process and during the experiment by wearing a suitable protective equipment.

## Methods

### Lead shield fabrication

The fabrication of the shield was using a high atomic number material (lead) and the thickness based on the gamma shielding calculation. Based on the energy produced from the Co^60^ source (1.3 MeV) with the dose delivery rate at 1.133 kGy/h, the lead shielding requires a thickness of 5 cm from the centre to reduce one-tenth of the dose rate. This shielding was molded into cuboid shape and covered the top of the mouse strainer except for the exposed area (1 cm × 1 cm) created to irradiate the tumour area precisely.

### Mouse holder

A mouse strainer was developed using Perspex with a dimension of 2.8 cm × 2.8 cm × 12 cm and placed inside the custom-built lead shield. An exposed area of 1 cm × 1 cm was prepared at the location aligned precisely to the beam path to enable beam entrance without scattered. During irradiation, a mouse holder was placed in an upright position with tissue paper at the edge. This holder stabilized the mouse at the right beam path for the beam entrance. The entrance of the canal of the lead shield is cover using a lead brick to avoid gamma-ray penetrate through the top of the lead shield.

### Cell line

EMT6 mouse mammary carcinoma cell line was purchased from the American Type Cell Culture (ATCC, USA). Cells were revived and grown in a T25 flask in a complete culture media that consist of Dulbecco’s modified Eagle’s medium (DMEM) (Gibco, USA), 10% Fetal Bovine Serum (FBS) (Gibco), penicillin G (100 IU/mL), and streptomycin (100 μg/mL) in a humidified incubator with 5% CO_2_ at 37 °C until 70–80% confluent.

### Mouse bearing tumour model

The mouse-bearing tumour model has been carried out according to Ibahim et al.^17^ and Yang et al.^18^. Briefly, female Balb/c mice age 6–8 weeks (weight 20–25 mg) (n = 12) were inoculated with EMT6 cells (100,000 cell/10 ul) in phosphate-buffered saline (PBS) subcutaneously on the right hind leg above the knee (stifle joint). Mice were irradiated at day-7 post inoculation where tumour volume less than 10% of body weight and were sacrifice 96 h post-irradiation by cervical dislocation. Mice were culled if the tumour-leg width reached 11 mm or showed symptoms of behavioural changes before the irradiation started. All procedures involving mice were conducted in accordance with relevant guidelines and regulations.by USDA Pain and Distress Categories that has been approved by the Universiti Teknologi MARA Animal Ethics Committee. Our procedure also adheres to the ARRIVE guideline to minimize the number of animals used, reduced the pain suffer to the animal and the person that handle all procedures is well-trained and approved to conduct the procedure.

### Gamma cell irradiation

Tumours were irradiated using a gamma-ray unit (Gamma Cell 220, Nordion, Ottawa, Canada) at the Faculty of Science and Technology, Universiti Kebangsaan Malaysia, Malaysia, at an operating dose rate of approximately 18.67 Gy/min. The gamma cell chamber is a vertical cylinder with 6-inch diameter and 8-inch height and is surrounded by a rod or pencil of the isotope. The chamber moved up and down, and the dose was delivered depending on the exposure time. Different calibration doses (0, 0.25, 0.5, 0.75, 1, 2, 4, 6, and 8 Gy) were delivered by adjusting the exposure times where the absorbed doses were measured using Gafchromic film EBT3. This film was attached to the mouse strainer and placed in the centre of the chamber. For lead shield dose measurement, a single dose of 30 Gy up to 90 Gy were delivered to achieve absorbed dose 2 Gy and 8 Gy (6 mice per group) based on preliminary experiment that used 8 Gy - 105 Gy. The chosen absorb doses were based on a previous study by Ibahim et al.^17^.

### EBT3 Gafchromic film

EBT3 Gafchromic film was purchased from Ashland Specialty Ingredient (USA) and cut into the dimensions of 2 cm × 2 cm and 7 cm × 1 cm for calibration dose and lead shield dose measurement, respectively. The film was attached to the cell strainer and transferred into a plastic bag for digitation.

### Film digitation and analysis

Film scanning was performed using an Epson Flatbed V600 scanner with a resolution of 300 dots per inch (DPI) using red, blue, and green (RGB) channel and saved as in Tagged Image file format (Tiff). The scanned images were analysed using ImageJ software and measured twice using RGB. The net reflective optical density (NetRod) was calculated using the pixel values generated by ImageJ using the formula Log (I_u_/I_i_) (Equation 1) where I_u_ was the average pixel value of unirradiated film and I_i_ was the irradiated film^19^. The calibration curve was constructed using the polynomial order three with an equation.

### Statistical analysis

Data are presented as mean ± standard deviation.

## Supplementary Information

Supplementary Figures.

## References

[1] Baskar R, Lee KA, Yeo R, Yeoh K-W (2012). Cancer and radiation therapy: Current advances and future directions. Int. J. Med. Sci..

[2] Barker HE, Paget JTE, Khan AA, Harrington KJ (2015). The tumour microenvironment after radiotherapy: Mechanisms of resistance and recurrence. Nat. Rev. Cancer..

[3] Demaria S, Golden EB, Formenti SC (2015). Role of local radiation therapy in cancer immunotherapy. JAMA Oncol..

[4] Weichselbaum RR, Liang H, Deng L, Fu Y-X (2017). Radiotherapy and immunotherapy: A beneficial liaison?. Nat. Rev. Clin. Oncol..

[5] Koontz BF, Verhaegen F, De Ruysscher D (2017). Tumour and normal tissue radiobiology in mouse models: How close are mice to mini-humans?. Br. J. Radiol..

[6] Ghita M, McMahon SJ, Thompson HF (2017). Small field dosimetry for the small animal radiotherapy research platform (SARRP). Radiat. Oncol..

[7] Gan GN, Altunbas C, Morton JJ (2016). Radiation dose uncertainty and correction for a mouse orthotopic and xenograft irradiation model. Int. J. Radiat. Biol..

[8] Yi L, Hu N, Yin J (2017). Up-regulation of calreticulin in mouse liver tissues after long-term irradiation with low-dose-rate gamma rays. PLoS ONE.

[9] Ghita M, Dunne V, Hanna GG, Prise KM, Williams JP, Butterworth KT (2019). Preclinical models of radiation-induced lung damage: challenges and opportunities for small animal radiotherapy. Br J Radiol..

[10] Schleich N, Herst P. Dosimetry for treating brain tumours in mice using a gamma irradiator. 2014 CSM EPOS. Published September 4, 2014. https://epos.myesr.org/poster/ranzcr/ranzcr2014/R-0293. Accessed 7 June 2021.

[11] Kaplon R, Hadziahmetovic M, Sommerfeld J (2013). The application of radiation therapy to the Pediatric Preclinical Testing Program (PPTP): results of a pilot study in rhabdomyosarcoma. Pediatr. Blood Cancer..

[12] Silva CR, Pereira ST, Napolitano CM, Somessari ER, Ribeiro MS (2020). Development of a shielding device for radiotherapy of breast cancer-bearing mice. Braz J Radiat Sci..

[13] Selvaraj J, Rhall G, Ibrahim M (2020). Custom-designed Small Animal focal iRradiation Jig (SARJ): design, manufacture and dosimetric evaluation. BJR|Open..

[14] Soliman K, Adili M, Alrushoud A (2019). Radiation dose verification of an X-ray based blood irradiator using EBT3 radiochromic films calibrated using Gamma Knife machine. Rep. Pract. Oncol. Radiother..

[15] Howard ME, Herman MG, Grams MP (2020). Methodology for radiochromic film analysis using FilmQA Pro and ImageJ. PLoS ONE.

[16] Najafi M, Geraily G, Shirazi A, Esfahani M, Teimouri J (2017). Analysis of Gafchromic EBT3 film calibration irradiated with gamma rays from different systems: Gamma Knife and Cobalt-60 unit. Med. Dosim. Off. J. Am. Assoc. Med. Dosim..

[17] Ibahim MJ, Yang Y, Crosbie JC (2015). Eosinophil-associated gene pathways but not eosinophil numbers are differentially regulated between synchrotron microbeam radiation treatment and synchrotron broad-beam treatment by 48 hours postirradiation. Radiat. Res..

[18] Yang Y, Swierczak A, Ibahim M (2019). Synchrotron microbeam radiotherapy evokes a different early tumor immunomodulatory response to conventional radiotherapy in EMT6.5 mammary tumors. Radiother. Oncol..

[19] Farah N, Francis Z, Abboud M (2014). Analysis of the EBT3 Gafchromic film irradiated with 6 MV photons and 6 MeV electrons using reflective mode scanners. Phys. Medica Eur. J. Med. Phys..

