# Supplementary material for: Development of custom lead shield and strainer for targeted irradiation for mice in the gamma cell chamber
Source: Sci Rep. 2021 Jul 15;11:14559. doi: 10.1038/s41598-021-93964-5 (PMC8282798; doi:10.1038/s41598-021-93964-5)
Supplement: Supplementary file 1 — Supplementary Figures. [file 41598_2021_93964_MOESM1_ESM.docx]

Development of Custom Lead Shield and Strainer for Targeted Irradiation for Mice in The Gamma Cell Chamber

# Nurhaslina Hasan1,2,, Nur Fatihah Ronny Sham1, Muhammad Khalis Abdul Karim3, Syed Baharom Syed Ahmad Fuad1^,^ Narimah Abdul Hamid Hasani1, Effat Omar1, Mohammad Johari Ibahim1,*.

1Faculty of Medicine, Universiti Teknologi MARA, Sungai Buloh 47200, Selangor, Malaysia.

2Faculty of Dentistry, Universiti Teknologi MARA, Sungai Buloh 47200, Selangor, Malaysia

3Faculty of Applied Science, Universiti Putra Malaysia, Serdang 43400, Selangor, Malaysia

[*mji@uitm.edu.my](mailto:*mji@uitm.edu.my)

Supplementary Figure

**
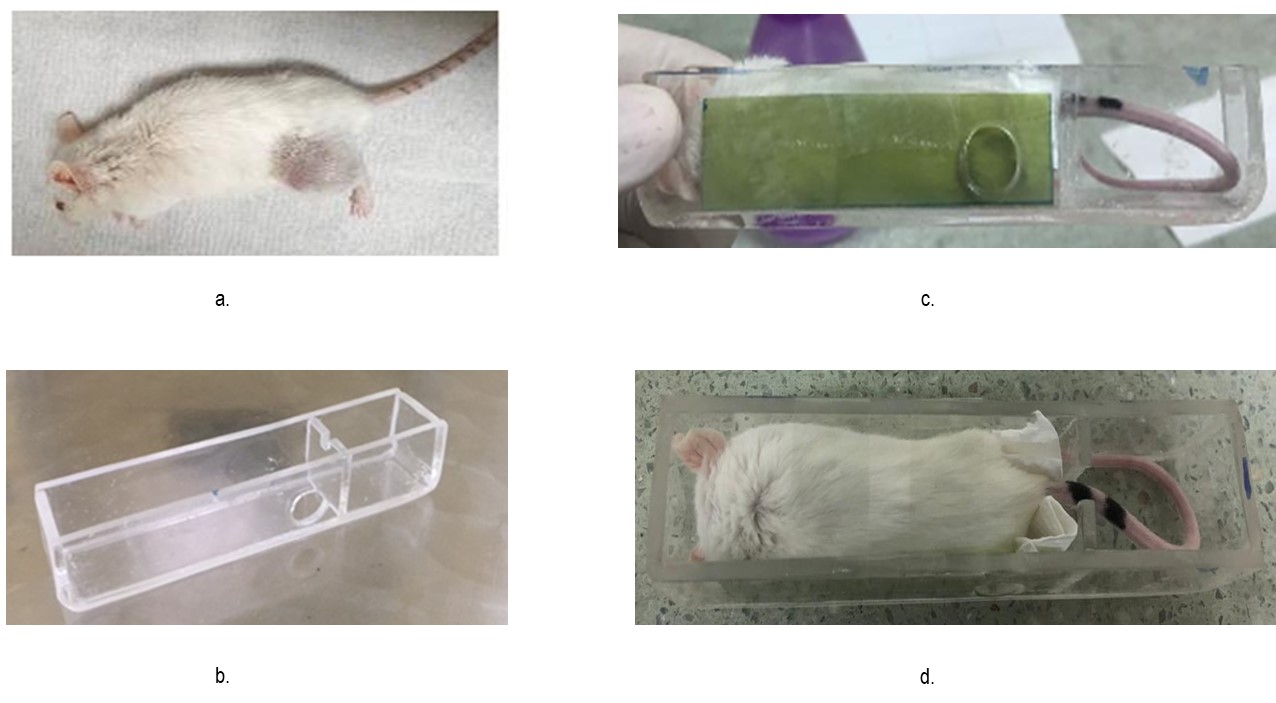
**

**Supplementary Figure S1**. (a) EMT6 mice bearing tumour model at hind leg after day 7 post-inoculation with tumour length approximately 7 mm. (b) The mice strainer. (c) and (d). The mice tumour position was adjusted directly to the hole of the strainer with EBT Gafchromic film was placed at the beam path (Top and side view).


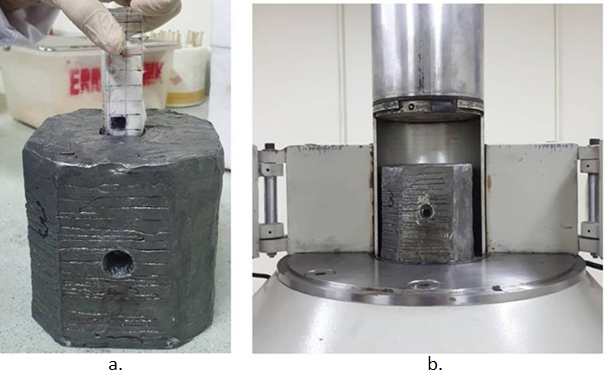


**Supplementary Figure S2.** (a) Mouse in the strainer was loaded in the lead shield canal. (b) The lead shield was transferred in the gamma cell chamber before irradiation.

**
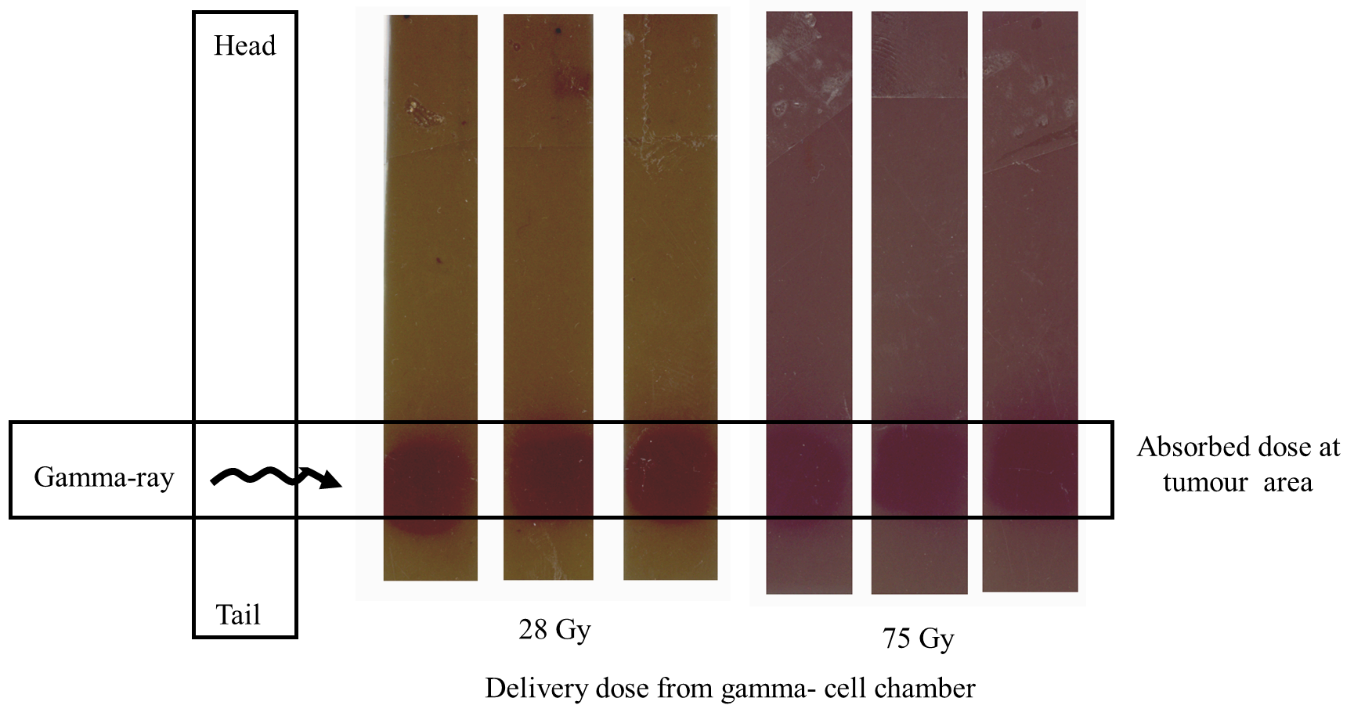
**

**Supplementary Figure S3**: The absorbed dose monitored using EBT3 Gafchromic film and calculated from the gamma-cell dose curve from 2 Gy to 105 Gy obtained from our preliminary work. The images show the film for 8 Gy and 75 Gy delivery doses.
